# Does the socioeconomic positioned neighbourhood matter? Norwegian adolescents’ perceptions of barriers and facilitators for physical activity

**DOI:** 10.1177/14034948211066673

**Published:** 2022-01-10

**Authors:** Hanne Hennig Havdal, Elisabeth Fosse, MEkdes Kebede Gebremariam, Karien Stronks, Oddbjørn Klomsten Andersen, Nanna Lien

**Affiliations:** 1 Department of Nutrition, Institute of Basic Medical Sciences, University of Oslo, Norway; 2 Department of Health Promotion and Development, University of Bergen, Norway; 3 Department of Nutrition, Institute of Basic Medical Sciences, University of Oslo, Norway; 4 Department of Public and Occupational Health, University of Amsterdam, Netherlands; 5 Department of Sports Medicine, Norwegian School of Sport Sciences, Norway; 6 Department of Nutrition, Institute of Basic Medical Sciences, University of Oslo, Norway

**Keywords:** adolescent, neighbourhood, physical activity, qualitative methodology, social inequality

## Abstract

**Background and aims:**

A higher proportion of adolescents from lower socioeconomic position families tend to be less physically active than their counterparts from higher socioeconomic position families. More research is needed to understand the causes of these differences, particularly the influence of the neighbourhood environment. This qualitative study aims to explore how adolescents and their parents from higher and lower socioeconomic neighbourhoods perceive the social, organisational and physical environment influencing adolescents’ physical activity behaviours.

**Method:**

We conducted six semi-structured focus groups with 35 13–14-year-olds and eight interviews with some of their parents. The interviewees were recruited from one higher and two lower socioeconomic neighbourhoods in Oslo, Norway. Theme-based coding was used for analysis, and the results discussed in light of an ecological framework.

**Results:**

The results indicate that factors like social norms in a neighbourhood could shape adolescents’ physical activity behaviour, and a social norm of an active lifestyle seemed to be an essential facilitator in the higher socioeconomic neighbourhood. Higher availability of physical activity and high parental engagement seemed to facilitate higher physical activity in this neighbourhood. In the lower socioeconomic neighbourhoods, the availability of local organised physical activity and volunteer engagement from parents varied. Programmes from the municipality and volunteer organisations seemed to influence and be essential for adolescents’ physical activity behaviour in these neighbourhoods.

**Conclusions:**

**The results illustrate the complexity of behaviour and environment interaction, and a limitation in explaining the phenomenon by focusing primarily on the individual level rather than an ecological perspective.**

## Introduction

Physical activity (PA) among adolescents has a beneficial impact on health-related quality of life [1] and overall health and weight [2,3]. Adolescence is defined as the time between the ages of 10 and 19 [4], and is considered a critical period for addressing energy-related behaviour such as PA [5]. For adolescents PA includes organised and unorganised leisure time physical activities (LTPAs). These are activities such as sports, exercising and recreational walking, which are not required as essential activities in their everyday living and are performed as a choice of the person [6]. Most adolescents are insufficiently active, and the proportion not meeting the recommendations of a minimum of 60 minutes of PA of moderate-to-vigorous intensity each day [7] increases with age [8-10]. Adolescents from families of lower socioeconomic position (SEP) tend to be less physically active than their counterparts from higher SEP families, both worldwide [11,12] and in Norway [8,13].

The individual’s SEP is usually determined through achieved educational level, occupation and/or income [14,15], and whether one looks at educational level or income [16], the higher the position, the better the health [14]. Levelling these inequalities is the aim of several policies and one of the main goals in the Norwegian Public Health Act [16,17]. Social inequalities are higher than expected in Norway compared with other European countries [18,19], and these are more present in Oslo with documented inequalities between the east and west side of the city [20,21]. A social gradient is found in PA levels among adolescents, particularly in organised sports participation [22,23]. Approximately 75% of adolescents in the higher SEP urban-districts in the western parts are regularly involved in LTPA, compared with around 45% of adolescents in the lower SEP urban-districts in the eastern parts [23]. Even though social inequalities are present in Oslo, the adolescents themselves, regardless of where they live, score relatively equally on loneliness, bullying, life quality, self-esteem, ailments, and how satisfied they are with their parents [23]. Hence, research on adolescents, SEP and LTPA needs to look beyond just individual behaviour to understand how environmental factors interact and influence LTPA [12,24,25].

Ecological models are suited to study LTPA and complex issues like social inequalities [26], and could increase understanding by focusing on the widely distributed causes that are not solely an individual responsibility instead of the victim-blaming ideology of harmful behaviours [27,28]. An ecological model could include five levels: intrapersonal, interpersonal/cultural/social (social level for the rest of the paper), organisational, the physical environment and policy [26].

Norwegian adolescents tend to spend more of their everyday life in their local neighbourhood than adults [29], and the environment they grow up in and its surroundings may influence life choices and possibilities, as is recognised in current strategies to level out social inequalities [15]. Neighbourhoods vary considerably across and within countries, and are a mix of social, physical and organisational structures [29]. Individuals living in the same environment, such as a neighbourhood, who experience the same surroundings or share the same socioeconomic background, tend to adopt similar behaviour within social or environmental patterns [30].

Research on social-level determinants associated with adolescents’ PA behaviour and SEP influence is limited and needs more attention [22,31]. Family resources, immigrant origin, and social norms from peers and parents can only partially explain these differences [22,31]. A qualitative study from Sweden of adolescents from multicultural, lower SEP neighbourhoods described parents’ nagging and demanding as PA barriers and desired parental support and engagement [32]. Existing research of adolescents’ PA behaviour and their built environment has mixed results [33], providing a knowledge gap due to large differences between environments and an unclear possible influence of SEP [25,33]. A systematic review of qualitative studies that explored adolescents’ perspectives on the barriers to and facilitators of PA stresses that much of the research either does not consider SEP in the analysis, or that only adolescents in higher or lower SEP neighbourhoods are presented [34]. Adolescents living in a lower SEP neighbourhood in Amsterdam expressed in a qualitative study that safety and distance to PA were perceived as environmental barriers for PA involvement [35]. Swedish adolescent girls in lower SEP neighbourhoods said that fewer PA opportunities for girls was a reason for not participating in organised PA [36].

Complementing the present mixed results on adolescents’ PA behaviour and social inequalities with qualitative research will provide more in-depth knowledge. Hence, this qualitative study aims to explore how adolescents, and some of their parents, from higher and lower SEP neighbourhoods in Oslo perceive their environment to influence adolescents’ LTPA behaviours. The environment includes the social, organisational and physical environments, and policy level in an ecological view. Specifically, we explore perceived barriers and facilitators that influence adolescents’ LTPA behaviour and whether these barriers and facilitators appear to differ by neighbourhood SEP. Special attention was given to the social level, including friends, family and the neighbourhood’s culture, and the organisational and physical environment levels, including organisation, availability and LTPA facilities in the neighbourhood. Last, we briefly explore the policy level, including plans and opportunities facilitated by the municipality.

## Methods

Before data collection, the Norwegian Centre for Research Data (NSD) approved the project’s data protection. All focus groups and interviews with parents started with a presentation of the project and the main topics, the project’s ethical principles (i.e. confidentiality, the interviewees’ rights and possibilities of leaving with no questions asked) and a space for questions [37,38]. Adolescent participation required signed informed consent from the parents, and the participating parents signed an informed consent form for the personal interviews.

### Recruitment of participants

As a divided city in terms of social inequalities [20,21], Oslo was considered a relevant place. To select diverse urban-districts from which more local neighbourhoods would be included, publicly available data on sociodemographic characteristics of the urban-districts of Oslo were explored [39]. The prioritised criteria were life expectancy, educational level, mean income, and adolescents’ participation in organisations, clubs, teams or associations.

Three areas were selected, reflecting diversity in SEP (Table I).

**Table I.** Selected statistical data describing three areas in the municipality of Oslo, Norway [39].

Areas in Oslo	Life expectancy (men) (years)	Lower educational level^a^ (<13 years) (%)	Mean income (NOK^b^)	Organisation participation – lower secondary school^c^ (%)
Northeast (three urban-districts)	77.4–80.2	67–73	361 000–384 000	43–48
Southeast (two urban-districts)	80.8–81	50–56	381 000–472 000	54–62
West (three urban-districts)	82.4–83.6	36–38	583 000–791 000	73–78

a 2016–2017: lower educational level was classified as having completed primary and lower secondary school (10 years), upper secondary school (13 years), or vocational training. Higher educational level was classified as having attended a university or university college (Bachelor’s, Master’s or higher).

b 2017: mean income in Oslo municipality, NOK 507 000.

c 2015: organisations, clubs, teams or associations.

In the three selected areas, west, south-east and north-east, 32 lower secondary schools were located [40]. By using Google maps, the researcher examined the schools’ surroundings, the neighbourhood and the activity facilities. Twelve schools had food and activity facilities surrounding the adolescents’ school within walking distance (~30 min) and were contacted for participation. Out of the twelve contacted schools, three responded positively to participation: one located in the north-east of Oslo, one in the south-east and one on the west side. At the schools, the 8th-grade students were invited, and written information letters with consent forms were provided for those interested in participating: one for the parents and an age-adjusted letter for the adolescents. The letter for the parents also included information and questions about participating in personal interviews.

Before conducting any interviews or focus groups, the researcher walked around in each of the three included neighbourhoods one time. This was done a few days before the focus groups interviews but did not follow a qualitative observational methodology. These observations provided the interviewer with a better understanding of the neighbourhoods and recognition of places mentioned by the interviewees, thus easing follow-up questions during the interviews.

#### Focus groups with adolescents

When recruiting the adolescents, we were interested in the adolescents’ perceptions of their local neighbourhood collectively as a focus group and not through their individual status. Personal information beyond gender, age and parental educational level were therefore not collected. In total, 35 adolescents, aged 13–14 years, agreed to participate, resulting in two focus groups at each school, six in total. In the consent form, parents reported the age and gender of their adolescent in response to open questions. In addition, parental educational level was collected to make sure the participants mirrored their neighbourhood. Education level varies between the sub-districts in Oslo (Table I) and is often used to determine SEP [14,15]. The parents were asked to tick the highest achieved educational level: primary school/lower secondary school, upper secondary school, vocational training or university/university college. These were grouped into categories of lower and higher educational level (Table II). Lower educational level includes primary and lower secondary school (10 years), upper secondary school (13 years) and vocational training. Higher educational level includes university and university college (Bachelor’s, Master’s or higher). The educational level of the adolescents’ parents mirrored their overall area statistics shown in Table I.

**Table II.** Description of adolescents in six focus groups by areas in Oslo, Norway and the educational level of their parents.

Area	Participants		Parental educational level^a,b^	
Northeast 1	4 boys	3 girls	9 lower	3 higher
Northeast 2	2 boys	4 girls	8 lower	4 higher
Southeast 3	2 boys	4 girls	4 lower	7 higher
Southeast 4		3 girls	5 lower	1 higher
West 5	2 boys	4 girls	2 lower	9 higher
West 6	2 boys	5 girls	2 lower	12 higher

a Lower educational level includes having completed primary and lower secondary school (10 years), upper secondary school (13 years) or vocational training. Higher educational level includes attending university or university college (Bachelor’s, Master’s or higher).

b Some adolescents only had parental educational level for one parent.

#### Interviews with parents

The present study includes interviews with some of the adolescents’ parents. Adolescence is a transition period to more independence from parents, while friends and peers typically become more influential [41]. Thus, the purpose of including parents was to get their view as being an important part of adolescents’ lives and to provide a broader picture of the neighbourhood and its possible influence. The interviews included eight individual parents, two men and six women, age range 40–65 years, with five having a higher educational and three a lower educational level (Table III). Seven parents were recruited through their children’s participation in the focus groups. In addition, one mother from the south-east neighbourhood was recruited through snowballing. Recruitment of parents with lower educational level was more challenging, and snowballing was therefore perceived as a reasonable strategy. The included mother had lower educational level, she lived in the same neighbourhood as the other parents and had a daughter in 8th grade at the included school who did not participate in the focus groups.

**Table III.** Description of parents participating in interviews by areas in Oslo, Norway: educational level, age and gender of participating child in the adolescent focus groups of the study.

Area	Participants	Age (years)	Parental educational level^a^	Adolescent
North-east	Mother 1	40	Lower	Daughter
	Father 1	61	Higher	Son
South-east	Mother 2	41	Higher	Son
	Mother 3	49	Lower	Daughter
	Mother 4	51	Lower	Daughter^b^
West	Mother 5	46	Higher	Son
	Mother 6	42	Higher	Daughter
	Father 2	65	Higher	Daughter

a Lower educational level includes having completed primary and lower secondary school (10 years), upper secondary school (13 years) or vocational training. Higher educational level includes attending university or university college (Bachelor’s, Master’s or higher).

b Not a participant in the focus groups.

### Materials

A semi-structured interview guide for the adolescents was developed. The main topics included questions about LTPA and opportunities in their neighbourhood, both organised and unorganised. There were also questions about parental engagement, adolescents’ engagement in organised LTPA, and facilitators for and barriers to being engaged in any LTPA. The interview guide was pre-tested for clarity, inputs and length with a gender-mixed group of five 8th graders. Based on the questions asked of the adolescents, a semi-structured interview guide for the parents was developed. The interview guide started with a section on dietary behaviour before LTPA. The first question on PA was one all could relate to: ‘picture a new girl or boy moving to this neighbourhood. What type of physical activity would you recommend or describe as the most frequently used in this neighbourhood?’. The main topics about LTPA included organised and unorganised LTPA opportunities in their neighbourhood, adolescents’ engagement in organised LTPA, parental engagement, and facilitators and barriers for engaging in LTPA in their neighbourhood.

Dietary behaviour was also covered during the interview, but these results are reported separately [42].

### Procedure

The focus group interviews with the adolescents were conducted at their school during school hours in March and April 2019. Interviews with the parents were conducted at a place and time of their choice in May 2019. The same moderator, with a background in public health nutrition, facilitated all interviews, which were audiotaped and lasted approximately 1 hour.

### Data analysis

All interviews were transcribed verbatim using the program f4transkript and were checked multiple times for accuracy and verification by listening to the interviews several times. The transcribing and analysis process started during data collection. The interviews with the parents and adolescents were analysed separately using the same analytic method. The analysis was performed using a descriptive approach of systematic text condensation through theme-based coding. The method aims to present the participants’ own experience expressed through their own words, rather than looking for other underlying meanings of what was said [43,44].

The method describes four analytic steps [43,44]: (1) obtaining an overall impression, (2) identifying meaning-units, (3) abstracting the content in the meaning-units and (4) summarising the overall meaning. All transcribed interviews were read thoroughly in the first step with an open mind and a bird’s-eye view. Preliminary themes that emerged from the text were noted and there was a high focus on the participants’ stories. In the second step, the interviews were read more systematically, and meaning-units were identified and coded. In this step, themes that were mentioned by several interviewees or repeatedly mentioned by participants in one area and not in others were noted. Also, new themes that appeared spontaneously from the interviews and were not covered by the interview guide were noted. Larger bits of text were highlighted in this process to ensure that no valuable text was discarded. In the third step, meaning-units were systematised, highlighted, and critically considered in light of the research questions. There was a continuous reviewing of the themes and meaning-units and returning to the original empirical materials for insurance and verifications. In the last step, re-contextualisation and text development of the findings were essential. A continual return to the original empirical material for assurance and verifications was important. HHH and EF read all interviews and had thorough discussions during all four steps providing validations of the findings.

## Results

The results from the focus groups and interviews of parents in the three neighbourhoods in Oslo, the higher SEP neighbourhood in the west, and the two lower SEP neighbourhoods in south-east and north-east, are presented as barriers and facilitators within an ecological framework. First, the themes at the social level are described before themes on the organisational and physical environmental level are presented. Last, a few results on the policy level are described. The two eastern neighbourhoods showed similarities, and these are therefore often presented together as ‘east’. To avoid an individual focus, the results are collectively presented, exemplified through individual voices.

### The social-level barriers to and facilitators of PA behaviour

#### Social norms

An essential facilitator in the higher SEP neighbourhood in the west seemed to be a social norm of an active lifestyle. The interviewed adolescents in this neighbourhood described how almost everybody they knew was involved in one or several organised LTPA. One boy said: ‘I can’t think of anybody who doesn’t do any physical activity’. The three interviewed parents confirmed this by describing the typical family from this area as an active one where the whole family is engaged in various sports activities depending on the season.

I think it’s kind of an image for a good west-side family to go cross-country skiing and downhill skiing and to be active in one way or another. Sailing in the summer, water skiing . . . people post pictures of their activities. It’s not like ‘in our family, we sleep till twelve every Saturday, and then we lie on the couch’. I am sure many people do, but that is not what you are posting on social media. Many people love to tell how active they are.

(Mother 5 – higher educational level, west)

The three parents in the west described how the adolescents in their neighbourhood showed engagement in being visible on social media through sports performances or portraying themselves as active and interesting through social media posts.

I think social media is a very important part . . . there is one girl who is very good at her sport. Posting pictures all the time and has almost become a mini celebrity at her school. But she’s like, she’s good at it. So it’s like, they have this need to show that they have talent, that they are good at things, and then it’s almost like they have to post it. [. . .] It is somewhat cool to look fit, and it is cool to show that you are exercising. Two of the girls are supposed to run every morning before school, and they don’t. But at least they make sure to take a picture of it [the times they do].

(Mother 6 – higher educational level, west)

In the focus group interviews, adolescents from the western higher SEP neighbourhood voiced how they juggled prioritising and organising their lives, including organised LTPA. Several of them said that being with friends was important, but this was often prioritised during the weekends when they could relax and have one day without any plans, just to be with friends and ‘chill out’. Some mentioned that they would never drop a sports practice to be with friends, and they would schedule their activity before their homework. However, the adolescents also talked about being stressed if they did not exercise.

Girl 4: I have to kind of exercise to feel good or to make the week work. Or to be pleased with the week. I kind of get stressed if I only work out once a week.

Boy 2: We tend to have a lot of homework at the beginning of the week. I exercise Monday, Tuesday and Thursday and exercise at home on Wednesday. So I’ve made a deal with my teachers that if I can’t do my homework, I do it the next day.

Girl 2: I don’t necessarily stress that much, but I like to have some sort of order knowing I’m doing things. And I exercise like every day . . . and it is a bit stressful sometimes. So on Mondays, I rarely do my homework but do it Tuesday morning.

(Focus group 5, west)

In the two eastern, lower SEP neighbourhoods, the perceptions and norms seemed different. When asked what most adolescents did after school hours, several described hanging around in the neighbourhood, visiting the mall or a fast-food restaurant, or just walking around. One boy in the south-east said: ‘Most people just hang with friends and walk around’. The interviewees in these two neighbourhoods did not describe engaging in organised LTPA, highly occupying themselves or organising everything around an activity in the same way as the adolescents in the higher SEP neighbourhood. On the other hand, some of the boys in the eastern, lower SEP neighbourhoods described how they could play unorganised football with friends, and the girls said they would often watch this. In the south-east neighbourhood, the adolescents described how fewer girls were involved in organised LTPA, and they felt it could be strange and awkward to start an activity alone with no other girls present.

#### Parental engagement

The adolescents in the higher SEP neighbourhood in west described parents in their neighbourhood as highly engaged and even too involved in the adolescents’ organised LTPA. At the same time, they liked having support from their parents. For example, one girl said, ‘Dad wants me to be . . . or I want to be good, so he helps me with that in a way’ – showing both the push and the support of the parental engagement. The adolescents also told stories during the focus groups interviews of angry parent-coaches and parents demanding too much.

In terms of choosing [a sport] favourably from a young age, some [parents] get pretty angry at the kids if they don’t perform.

(Boy 1, focus group 6, west)

The three interviewed parents in the higher SEP neighbourhood described how many parents in their neighbourhood had a high focus on and expectation of their child’s performance and dedication to their activities. They would often document and share their children’s performance on, for example, social media, showing other parents their active lives as a family and their children’s accomplishments.

I think a lot of parents are concerned that their children should do something, and some are also focused on how the children should perform and show it off. Post movies or talk about their kids being good.

(Mother 5 – higher educational level, west)

The adolescents in the two lower SEP neighbourhoods in the east did not mention parental engagement to the same degree as the adolescents in the higher SEP neighbourhood. It varied and was challenging to grasp the engagement. Some of the adolescents described how parents could drive them to and watch matches but were not directly involved; others described the influence of the cultural background.

Girl 3: My parents are quite cultural, so they were a bit sceptical when I said I wanted to start boxing, it’s a boys’ sport, it’s not a girls’ sport. But I persuaded them, so now they are okay with that.

Girl 2: Dad loved it when I wanted to start boxing

Boy 1: My mother supports me a lot in sports and such, but my father thinks that sports are . . . he likes football, but other sports he thinks are just nonsense and that I just should focus on school and such

(Focus group 2, north-east)

The parents in the two lower SEP neighbourhoods described some of the same perceptions and said that generally, only a few parents in their neighbourhood were engaged, although those who did were highly involved. The mother from the south-east thought of several reasons for this, such as cultural aspects, work hours or not having a driving licence. The father from the north-east almost felt unique through his involvement.

Interviewer: Do you have any thoughts about who the committed parents are?

Mother 2: Ehh, yes . . . the committed parents are from all social classes, but . . . But then you do have parents who work a lot at night. And some have many children, and that makes it not possible for them. So it might be that they are engaged at home, but they cannot be present at matches and such. And the challenge that they may not have a driver’s licence or do not have a car or . . . and then you cannot drive . . . so I think there is a lot that can influence

(Mother 2 – higher educational level, south-east)

Interviewer: At football games and such, are there a lot of parents?

Father1: There are some parents, yes . . . but not so many. It’s not. . . it’s like I said, they buy equipment for them [the children], new equipment, and give them a football and say please play, but nothing more.

Interviewer: Do you think parents feel pressure to get involved?

Father1: Yeah . . . it’s true. . .when you get involved, it means you have a responsibility and people do not want to take responsibility for others. But I have taken responsibility for other children, I have driven them home in the evening, here and there. I spent my own money. The others thank me, but. . .I do not give up. Because I do not want to be the same as them . . . So I try to be a bit. . . not unique, but I do it because I like it.

(Father 1 – higher educational level, north-east)

#### Becoming the best at an early age

Dividing adolescents into different teams, classified by skills and performance, so-called A- and B-teams, was eagerly discussed in the western, higher SEP neighbourhood’s two focus groups. This was not mentioned in the eastern, lower SEP neighbourhoods. The adolescents in the higher SEP neighbourhood discussed how this could be a facilitator for the good ones, but a barrier for those who only want to exercise without competing or becoming a champion. At the age of 13–14, the adolescents felt that they had to make that choice.

Boy 2: Having team A and B sucks.

Boy 1: I agree that it is nonsense, but we are starting to reach an age where I think you should be able to cope with a bit more discrimination in terms of skill level. I am a bit more in agreement with that now than I was a couple of years ago.

Girl 1: Guess it is fun for those who are interested in it. Even if you think an activity is fun, it doesn’t mean you want to compete in it all the time. There are several clubs that offer only exercise now, where no one is saying you should compete or anything and you can just be present at the training. Because not everyone wants to become the best. And it is a bit tricky if you end up with a team where everyone is really interested, and everyone thinks they should exercise to become world champions.

(Focus group 6, west)

One barrier mentioned in the higher SEP neighbourhood in the west was the challenge to change or start a new organised LTPA after the age of 10–14. This was grounded in the sports clubs focusing on forming talents at an early age, starting at a beginner’s level with a younger group, and feeling insecure about their own abilities.

I would think that to start with a new sport when you are 13–14 years old, it is a bit vulnerable because they have just become good, or almost, and started to perfecting. Because the worst thing she knows is to feel silly or dumb in a way, and then it might be challenging to get into an established environment.

(Mother 2 –high educational level, west)

#### Labelling

As the adolescents in the two lower SEP neighbourhoods described a general attitude of not being engaged in an organised LTPA, engagement barriers were explored further. The adolescents in the north-east neighbourhood mentioned how they could be labelled or bullied if they participated in an organised LTPA that was not socially accepted. For example, the girls in this neighbourhood described how they often would not wear shorts in the summer even though they wanted to, because they could get labelled as ‘whores’. Both boys and girls could be bullied for their chosen activity.

Boy 2: I actually wanted to go horseback riding before, I really wanted to, but I was a bit scared that people would call me . . . gay and stuff . . . This neighbourhood has evolved . . . there has become more violence and stuff like that. Football is a good activity, but horseback riding . . .

Girl 4: But it has become more common for boys to go dancing now, it’s not as big a deal as it was before.

Boy 1: But they are often bullied.

Boy 2: For guys, it fits with hip hop and dance like that.

Girl 1: If they dance any other dances they are seen as gaa . . . [cannot say the word].

Boy 1: Gay.

Interviewer: Can girls be bullied for some sports?

Several: Yes.

Boy 1: Yes, they can be called . . . I shouldn’t say that . . . but . . .

Interviewer: You are allowed to say what you want.

Boy 1: Okay, they can sometimes be called transvestites. If a girl really likes football and plays, they would say like . . . what’s with those girls. You’re a trance!

(Focus group 2, north-east)

### The physical level barriers to and facilitators of PA behaviour

#### Unorganised activities

The researcher’s brief observations when walking around in the three included neighbourhoods showed how all neighbourhoods had the forest surrounding Oslo close by with opportunities for outdoor recreation and unorganised activities. The higher SEP neighbourhood in the west was dominated by detached houses often accompanied by extensive gardens, many of them containing trampolines, and the school was surrounded by a green lawn. In the lower SEP neighbourhoods in the east, grassy open spaces or football fields surrounded the two included schools. Especially the northeast lower SEP neighbourhood had facilities for several different types of outdoor activities.

Parents in all three neighbourhoods perceived adolescents to be generally sedentary. The interviewed parents in the higher SEP neighbourhood in west talked about how they never saw children playing in the streets or using the neighbourhood for unorganised outdoor activities like they used to do when they were young. The adolescents in this neighbourhood did not describe any unorganised activity as part of their everyday lives.

We did have basketball goals here and we will hang them up again. So they can play some ball and such. So there is some activity. But not like in my childhood, it was probably more common to meet outside in the neighbourhood and play. It was a lot of fun right. It is very organised today, and it takes some of that creativity away. It means that once you have some spare time, you may want to do something other than exercise. You do so much exercise already.

(Father 2 – high education, west)

In the lower SEP neighbourhoods in the east, the interviewed parents described how adolescents in their neighbourhood occasionally used the football fields to meet and play. This was also expressed by the adolescents themselves and especially the boys.

#### Diverse availability of organised sport

In the higher SEP neighbourhood in the west, the absence of unorganised activity was in contrast to the topic of organised LTPA. The three parents and adolescents said they found it hard to think of one organised sport that was unavailable in their neighbourhood or other nearby urban-districts easily accessible by public transport.

In the two lower neighbourhoods, the adolescents mentioned several organised sports options, but fewer opportunities for organised teams for girls and differences in engagement between boys and girls. If girls in these neighbourhoods wanted to practise sports activities such as football or handball, they would often have to travel to other urban-districts, which several said they felt was too far and they would have preferred activities in their local neighbourhood.

Girl 1: I know it’s really important to be active, but it’s a bit difficult. But there is so little variety for girls here in the neighbourhood.

Girl 3: That’s so true.

Girl 1: I started on handball, but then they moved to a different borough, and it was a bit like . . . you have to take transport to get there, so it will be a bit difficult. Because here, you could just walk.

Girl 2: It makes it a bit easier here . . .

(Focus group 3, south-east)

#### Programmes from the municipality

In the two lower SEP neighbourhoods, the interviewed parents said that Oslo municipality had provided programmes for the urban-districts to help the neighbourhoods, through facilities, volunteers and opportunities, to have a more active lifestyle. The programme was still running in the north-east neighbourhood, providing the neighbourhood with different facilities, whereas it was stopped in the south-east neighbourhood.

Mother 3: The urban-district had such a programme where volunteers played football with them in the sports arena. So it was very good. They were there for 2 years. And all the young people were there every Friday, and then they had a match, and they trained. It was voluntary.

Interviewer: But do you have any thoughts as to why so many young people are not engaged in physical activity around here?

Mother 3: Had there been better offers and engagement with the help of the school, for example. That would have been very nice. Some voluntary effort. Or the urban-district, for example.

(Mother 3 – lower educational level, south-east)

The parents in the lower SEP neighbourhoods felt that these programmes had made a difference in facilitating organised LTPA and making the environment more available for activity. For example, in the north-east neighbourhood, the interviewed adolescents and the two parents talked eagerly about how many football fields they had and how they could borrow sports equipment for free from the municipality. This was especially important for the winter activities such as cross-country or downhill skiing, since a lot of the adolescents in this neighbourhood did not own a pair of skis.

Boy 2: If you have registered, you can borrow skis. Or if you have created a profile. And it’s free and you can just return them.

Interviewer: Are there many who use this?

Boy 4: Yes, my friends do at least.

Boy 2: I only did go once and I fell. So I quit.

Boy 4 I did too, and I was about to give up, but I did it again, and I managed.

(Focus group 1, north-east)

## Discussion

This qualitative study’s results show how several factors could influence PA behaviour separately and interact with each other. The discussion structure follows the themes from the results, and presents the interactions at different levels in an ecological model. We primarily focus on the social, organisational and physical environment, but do also include the policy levels.

In Oslo, around 73% of children aged between 0 and 17 years live together with two parents, while 20% only live with one parent. Around 19% of families have one or two children, while only 3% have three or more children. Both parents are typically working, as almost 70% of the inhabitants aged 15–70 years in Oslo are employed, though fewer in the eastern part of the city [39]. Norwegian culture as expressed through outdoor recreation activities such as hiking and skiing is robust, and reflected in the expression: ‘Norwegians are born with their skis on’. These activities are often executed during weekends or holidays, and symbolise the Norwegian identity and family tradition for many [45,46].

The neighbourhoods’ social norms seemed to shape adolescents’ PA behaviour, and several factors appeared to interact with these norms. The social norms expressed in the higher SEP neighbourhood in the west could be driven by the described cultural norms in Norway of an active lifestyle. Factors like portraying a dedication to organised LTPA, the pressure of becoming the best, and organising and juggling their lives to include homework, LTPA and friends are essential. These norms of an active lifestyle were also expressed through the parents’ comments of portraying a healthy family. For example, skiing together as a family reflects the typical family from the west side of Oslo.

The lower SEP neighbourhoods in the east were described as multicultural, and the typical Norwegian culture of outdoor recreational activities could thus be less normative. For many adolescents, outdoor activities such as skiing or hiking in the woods might need to be learned, and a recent report encourages municipalities to focus on this in their work in reducing social inequalities in PA [47]. Thus, resolving a PA barrier could require changes on different levels of the ecological model simultaneously. It could be detected on the social level, but behavioural changes require policy actions concerning opportunities to learn activities in the municipalities.

The norms could also be interpreted in light of the mentioned barriers in the lower SEP neighbourhoods in the east, like bullying and labelling, that appeared to lower social acceptance of LTPA, hence lowering the participation in LTPA.

The young generation is often referred to as ‘Generation Achievement’, causing several to feel pressure to perform on, for example, social media and in organised LTPA [48]. Social media can be challenging for adolescents, contributing to lower self-esteem, whereas using it to self-portray and peer-identify might positively support PA behaviour [49]. Around half of the Norwegian 15-year-olds spend 2 hours or more daily on social media [9]. In our study, social media seemed to be one of the factors driving the social norms of an active lifestyle in the higher SEP neighbourhood in the west by providing a platform for continually sharing results and performances. This could, of course, also be an essential factor in the lower SEP neighbourhood and might transcend some of the neighbourhood differences, but in our study, the adolescents and parents in the lower SEP neighbourhoods did not describe the use of social media as a driver for LTPA.

Social media has become a central part of our environment through communication, information sharing and marketing. Considering the social-level findings, this could be a platform for municipalities, volunteers, etc., which would be found at the highest level in the ecological model. Providing targeted information about LTPA options, increasing the focus on having fun and less pressure on becoming the best could be essential for adolescents from both higher and lower SEP neighbourhoods, and could be an initiative that targets all groups, as recommended, to level PA inequalities [15].

The availability of organised LTPA is on the physical environmental level in the ecological model. However, the results from this study indicate that availability could be influenced by other levels and factors in the model. The first is the influence of the norms on the social level. In the higher SEP neighbourhood in the west, the high availability of organised LTPA options seemed to be influenced by the norms for an active lifestyle that consequently could lead to a higher demand for a broad spectrum of LTPA opportunities in the neighbourhood. Several of the largest sports clubs offering skiing lessons or football are located in the higher SEP urban-districts on the west side of Oslo [50]. The mean income is higher, making economic barriers for attending organised LTPA fewer. As a result, being physically active is more feasible for adolescents in these parts of the city.

Another finding indicating the need to explore several levels in an ecological model simultaneously is found in the lower SEP neighbourhoods in the east, and how girls provided the impression of being less involved in organised LTPA due to several barriers they expressed as present in their local neighbourhood. In other research, girls have tended to be somewhat less active than boys in sports, and girls with a minority background are more often than other adolescents less active and never having participated in organised LTPA during childhood [8]. Barriers, such as expected voluntary parental engagement, economic resources, prioritising school work, and cultural or religious clothing, have been mentioned in previous research [51,52]. Our study indicated barriers at the social level in the ecological model expressed by the girls in these two neighbourhoods through variation in parental approval for activities, labelling from peers and clothing options. Barriers connected to organised LTPA availability in their local neighbourhood and parental engagement were found at the organisational and physical environmental level in the ecological model. This exemplifies how barriers could interact on several levels. For example, when girls perceive social barriers and consequently do not participate in organised LTPA, this could interact with the organisational and physical environmental level by leading to fewer organised LTPA options. Thus the involvement of volunteers and programmes from the municipality could perhaps help adolescents, especially girls, attend organised LTPA in these neighbourhoods.

The availability of organised LTPA at the ecological model’s organisational and physical environmental levels is also closely connected to parental engagement at the social level in the ecological model. In Norway, the organisation of organised LTPA is highly dependent on volunteer participation and engagement from parents, and usually takes place outside school. This differs from other countries and may vary locally across neighbourhoods, urban-districts and municipalities [53]. Parents are perceived to be essential for support in adolescents’ organised LTPA [54] while, on the other hand, youth LTPA is suggested to be too organised, serious and expensive, and demands more from parents, which again could lead to a culture of exclusion [55]. Participating in organised LTPA is more challenging if you do not have parental support, and it is challenging to have a football team in the neighbourhood without parental involvement. In this study, parental engagement as a phenomenon in the neighbourhood was the focus, and not the direct connection between one parent and his/her daughter/son. In the lower SEP neighbourhoods in the east, the parental engagement varied and the present study suggests that parental engagement could be influenced by several aspects and possible barriers such as family, religious and work situations. Therefore, the described support from volunteers and municipalities, located at the policy level in an ecological model, could be important and make a difference to involvement in these neighbourhoods. The mentioned services that lend out sports equipment are often run by the municipalities and/or volunteers to provide youth with opportunities to try different activities and even out social inequalities [56]. These municipalities’ or volunteers’ services or programmes could counteract the high costs of many LTPA activities that lead to exclusion from youth LTPA. These programmes or facilities could be essential for low-income families, numerous children or children attending several organised LTPAs [55].

### Strengths and limitations

A strength of this study is the inclusion of parents as well as adolescents from diverse neighbourhoods. By including parents, the data were enriched through their reflections, placing the neighbourhood in a bigger context. The included adolescents and their parents mirrored the population in the area where they lived satisfactorily, based on information about parental educational level. Still, the presence of a few more parents with lower educational levels could have provided the study with richer data. In general, it could be questioned whether only involved and engaged interviewees participated. Still, the interviewees were considered to be sincere and spoke openly about both barriers and facilitators.

As described by Malterud [44], data saturation was considered to have been met in the focus groups because there was an exhausting and no new data supported refinement of categories in the information between the two groups within each neighbourhood. Data saturation was also considered to have been met with the interviewed parents, but no parents with a lower educational level were interviewed in the higher SEP neighbourhood, representing a limitation of the data.

Focus groups, as a method, have been considered valuable with adolescents aged 11–14 years if discussions target personal experiences [57]. As adolescents can be vulnerable, especially in the meeting of a grown-up, authoritative researcher, all ethical principles were specified thoroughly, making sure everybody knew their rights and options with regard to the ethical principles [37,38]. The moderator was aware of the position as a researcher in public health, but the interviews were considered not to be affected by this as the interviewees talked freely about both healthy and unhealthy behaviour. Adolescents could be uncomfortable in revealing information, or the groups’ dynamics could be influenced by peers or gender mix [57]. This was carefully considered and discussed after the test-interview with a gender-mixed group and was not considered an issue during the interviews, but rather a strength.

The use of a semi-structured interview guide allowed the list of topics and questions to arise naturally and provided the moderator with some flexibility. Most of the included adolescents talked enthusiastically and had more to say than time was given, so the focus group was considered to be a suitable method.

Even though one moderator and one assistant are recommended for focus groups [58], the project did not have the resources for this. Therefore, detailed field notes of the interviews were written immediately after finishing to make sure as much information as possible was captured. The moderator also described all non-verbal body language verbally during the interviews: ‘you two are nodding’, ‘you are laughing’. Nevertheless, this could be considered a limitation.

## Conclusion

The present study results indicate that the perceived barriers and facilitators for LTPA varied between the socioeconomic neighbourhoods and that several factors could affect adolescents’ PA levels. Factors such as social norms in a neighbourhood could shape adolescents’ PA behaviour, and a social norm of an active lifestyle seemed to be an essential facilitator in the higher SEP neighbourhood. Higher availability and high parental engagement did also seem to facilitate higher PA in this neighbourhood. In the lower SEP neighbourhoods, the availability of local organised PA and volunteer participation and engagement from parents varied. Programmes from the municipality and volunteers seemed to influence and be essential for adolescents’ PA behaviour in these neighbourhoods.

The results illustrate a limitation in explaining the phenomenon if the focus is primarily at the individual level in an ecological model and not at several levels at the same time. The results indicate that adolescents in the different neighbourhoods could need different facilitation for LTPA. Research into this could be helpful and provide targeted measures at all levels to increase adolescent PA levels. Thus the results could inspire policy actions aiming to promote and facilitate PA behaviour for adolescents in their local neighbourhoods. The findings could be used to develop interventions and hypotheses for further research, focusing especially on the lower SEP neighbourhoods and opportunities for organised LTPA for all adolescents.
